# Immuno-Ultrastructural Localization and Putative Multiplication Sites of Huanglongbing Bacterium in Asian Citrus Psyllid *Diaphorina citri*

**DOI:** 10.3390/insects10120422

**Published:** 2019-11-23

**Authors:** El-Desouky Ammar, Diann Achor, Amit Levy

**Affiliations:** 1 Agriculture Research Service, -United States Department of Agriculture (ARS-USDA), Fort Pierce, FL 34945, USA; desoukyammar@gmail.com; 2 Citrus Research and Education Center, University of Florida, Lake Alfred, FL 33850, USA; dsar@ufl.edu; 3 Department of Plant Pathology, University of Florida, Gainesville, FL 32611, USA

**Keywords:** citrus, Hunaglongbing, Liberibacter, psyllid, TEM

## Abstract

Huanglongbing, the most destructive citrus disease worldwide, is caused by the bacterium ‘*Candidatus* Liberibacter asiaticus’ (CLas) and is vectored by the Asian citrus psyllid (ACP). Very little is known about the form and distribution of CLas in infected psyllids, especially at the ultrastructural level. Here, we examined these aspects by transmission electron microscopy, combined with immunogold labeling. In CLas-exposed ACP adults, the CLas bacterial cells were found to be pleomorphic taking tubular, spherical, or flask-shaped forms, some of which seemed to divide further. Small or large aggregates of CLas were found in vacuolated cytoplasmic pockets of most ACP organs and tissues examined, including the midgut, filter chamber, hindgut, Malpighian tubules, and secretory cells of the salivary glands, in addition to fat tissues, epidermis, muscle, hemocytes, neural tissues, bacteriome, and walls of the female spermatheca and oviduct. Large aggregates of CLas were found outside the midgut within the filter chamber and between the sublayers of the basal lamina of the hindgut and Malpighian tubules. Novel intracytoplasmic structures that we hypothesized as ‘putative CLas multiplication sites’ were found in the cells of the midgut, salivary glands, and other tissues in CLas-exposed ACP. These structures, characterized by containing a granular matrix and closely packed bacterial cells, were unbound by membranes and were frequently associated with rough endoplasmic reticulum. Our results point to the close association between CLas and its psyllid vector, and provide support for a circulative-propagative mode of transmission.

## 1. Introduction

Huanglongbing (HLB, also known as citrus greening)**,** the most destructive disease to citrus plants in the world, is putatively caused in Asia and the Americas by the phloem-restricted gram-negative bacteria *Candidatus* Liberibacter asiaticus (CLas), vectored by the Asian citrus psyllid (ACP, *Diaphorina citri*, Hemiptera: Liviidae) [1,2]. Current management options for HLB are limited and heavily rely on the application of chemical insecticides for limiting ACP vector populations. However, these strategies are challenging because of application and environmental problems and the development of insecticide resistance among psyllid populations [3]. A better understanding of the transmission process of CLas by its psyllid vector and the molecular mechanisms that underlay this process, could result in alternate management practices against the HLB disease.

CLas is an obligate pathogen that lives inside both the plant and the insect hosts. In the plant, CLas localizes in the phloem tissue, and is taken up by *D. citri* when the insect feeds. After feeding, it is hypothesized that CLas moves throughout the insect body in a circulative-propagative manner [4,5,6]. Transmission of CLas depends on the ability of the bacteria to multiply within the insect tissue and on its ability to cross barriers during the transmission pathway especially the gut-hemolymph and the hemolymph-salivary glands barriers. *D. citri* adults that acquired CLas as nymphs can remain infective throughout their life, and it was shown that they can transmit CLas much better than those that acquire the bacteria as adults [4,7]. This difference might result from the longer phloem feeding periods of nymphs compared to adults [8]. It was shown that CLas also propagates more efficiently in the nymphal stages of ACP compared to the adults, and this can also contribute to the higher transmission efficiency of ACP that acquire CLas as nymphs [4,7]. Differences in the innate immunity between *D. citri* nymphs and adults have been suggested, based on quantitative proteomic and microscopic studies of healthy and CLas-infected ACP [9,10]. Understanding the interactions between *D. citri* and CLas at the cellular and subcellular levels is important to our understanding of pathogen-vector interactions. Ultrastructural localization of *Candidatus* Liberibacter solanacearum (CLso), another member of the genus *Ca.* Liberibacter that cause diseases in solanaceous and umbelliferous crops, was studied in the potato psyllid *Bactericera cockerelli*. Results showing that the CLso bacteria were present in various organs including the gut and the salivary gland, pointed to a systemic invasion of potato psyllids by CLso and supported a propagative, circulative mode of transmission [11]. The anatomy of *D. citri* has been studied earlier by Ammar et al. and Cicero et al. [12,13]. The ultrastructure of the salivary glands, filter chamber, alimentary canal, compound ganglionic mass (in the thorax), and the bacteriome (in the abdomen) has been described, but without the presence of CLas cells [12,13]. Some early investigations reported that bacteria-like structures assumed huanglongbing pathogens in *D. citri*, without any supporting immuno- or molecular-labeling evidence [14,15]. Using qPCR and fluorescence in situ hybridization (FISH), the presence of CLas genome was detected in various psyllid organs [5,6], suggesting a systemic infection. Recently, Ghanim et al. [16] showed that in CLas infected *D. citri* guts, CLas accumulates inside Liberibacter containing vacuoles (LCVs), and that these vacuoles associate with the host cell rough endoplasmic reticulum (RER), suggesting that CLas can accumulate intracellularly in the psyllid cells. However, whether this occurs in cells other than those in the midgut is still unknown.

Here, we used transmission electron microscopy (TEM) and immunogold labeling to study the ultrastructural morphology and localization of CLas, inside different organs and tissues of infected ACP. Our results showed that CLas is abundantly found in tubular/filamentous and quasispherical forms in many different ACP organs and tissues, indicating a systemic infection. Putative CLas multiplications sites (associated with RER) in the cytoplasm of infected cells are also described. Our results point to the close cellular and subcellular association between CLas and its psyllid vector, and provide a strong support for a circulative-propagative mode of CLas transmission by ACP.

## 2. Materials and Methods

### 2.1. Psyllids and Plants Used

CLas-exposed adult psyllids (*D. citri*), fixed on 15 May 2017, were reared at CREC (Lake Alfred, FL, USA) for several generations (from egg to adult stages) on CLas-infected citrus plants (*Citrus macrophylla*) showing HLB symptoms, which proved CLas-positive in qPCR tests. Another batch of CLas-exposed adult psyllids, fixed on 25 April 2018, were reared at the ARS-USDA Lab (Fort Pierce, FL, USA) for several generations on CLas-infected citron (*Citrus medica*) that proved CLas-positive in qPCR tests. Healthy control psyllid adults (*D. citri*, non-exposed to CLas), fixed on 15 May 2017 and 25 March 2018, were reared at CREC (Lake Alfred, FL, USA) on healthy curry leaf plants (*Murraya koenigii*) which are known to be resistant to CLas infection. Monthly samples were analyzed by qPCR from the psyllid adults of each colony, with the percentage of CLas-positive adults (with average Ct values less than 30) fluctuating usually from 30%–100% in CLas exposed colonies, and 0% in the healthy control colonies. Psyllid rearing conditions and the qPCR procedures followed were as described by Ghanim et al. [16] for the CREC colonies and by Ammar et al. [17] for the ARS-USDA colonies.

### 2.2. Transmission Electron Microscopy (TEM)

Two different fixation procedures were used for TEM of adult psyllids (*D. citri*)—non-osmicated and osmicated. The first procedure was necessary for immunogold labeling of CLas cells in thin sections, and the second, for better preservation of the psyllids’ ultrastructural details.

Non-osmicated samples: Adult psyllids were anesthetized with acetone, then partially micro-dissected by removal of the wings, legs, and posterior third of the abdomen, for A better penetration of fixative and other reagents. These psyllids were fixed overnight in Karnovsky’s fixative, at 4 °C, then washed in Sorenson’s phosphate buffer (pH 7.2), dehydrated in graded ethanol over 2 h then infiltrated with LR White resin over 3 days. The samples were then embedded and polymerized in 100% LR White. One micrometer (semi-thin) sections were made, stained with methylene blue/azure A and basic fuchsin [18], and examined with light microscopy (LM). Some of these psyllids were serial sectioned for LM, and three grids with ultrathin sections (for TEM) were made for every slide of 20 one-micrometer thick sections. To help in the identification of various psyllid organs and tissues examined, the semi-thin sections were examined with an Olympus BX61 compound microscope (Cambridge Scientific Products, Watertown, MA, USA). Ultrathin sections of identified organs were stained with 2% aqueous uranyl acetate, followed by Reynold’s lead citrate [19], and were examined with a Morgagni 268 transmission electron microscope (FEI, Hillsboro, OR, USA). From the non-osmicated samples, 11 CLas-exposed and 6 healthy control psyllids were sectioned and examined by LM and TEM.

Osmicated samples: Psyllid adults were separated according to sex, anesthetized with acetone, and partially micro-dissected by the removal of legs, wings, and lower abdomen. These psyllids were fixed in 3% glutaraldehyde in Sorensen’s buffer overnight at 4 °C, washed, and post-fixed for 4 h, at room temperature in 2% osmium tetroxide in Sorenson’s buffer. The samples were dehydrated in either ethanol or acetone, infiltrated, and embedded with either LR White resin or Spurr’s resin [20].

Some adult psyllids were prepared for LM and TEM, as above, except that the psyllids were dissected by the separation at the head, thorax, or abdomen to further facilitate quick access to the various organs. Of the osmicated samples, four healthy control and 13 CLas-exposed psyllid adults were sectioned and examined by LM and TEM.

### 2.3. Gold Labeling of CLas Cells in Thin Sections

Thin sections of non-osmicated, glutaraldehyde fixed, LR White embedded CLas-exposed psyllids were made and mounted on nickel grids. The grids were floated for 15 min on 0.1% sodium borohydride in phosphate-buffered saline (PBS) (1× phosphate buffered saline), for aldehyde inactivation, followed by PBS rinsing on 4 drops of PBS, 10 min each. These grids were then floated for 30 min on drops of phosphate-buffered saline with tween (PBST) (1× phosphate buffered saline, 0.1% triton X) + 1% bovine serum albumin (BSA) (for permeabilization with the triton X and blocking with the BSA). The grids were then rinsed on three drops of PBS for 5 min each. They were then incubated for 2 h on primary antibody 1:100 (polyclonal antibody to an outer membrane peptide of Liberibacter (anti omp-pab) prepared by Abnova and sold by Fisher Scientific). The grids were then rinsed 3× on PBST + BSA, 5 min for each drop, then incubated for 30 min in 1:100 10 nm goat–antirabbit gold in PBS. They were then rinsed in a stream of PBS, followed by a stream of distilled water. The sections were then stained in uranyl acetate and lead citrate as described above.

## 3. Results

### 3.1. Ultrastructure and Immunolabeling of CLas Bacterial Cells

**Figure 1:**
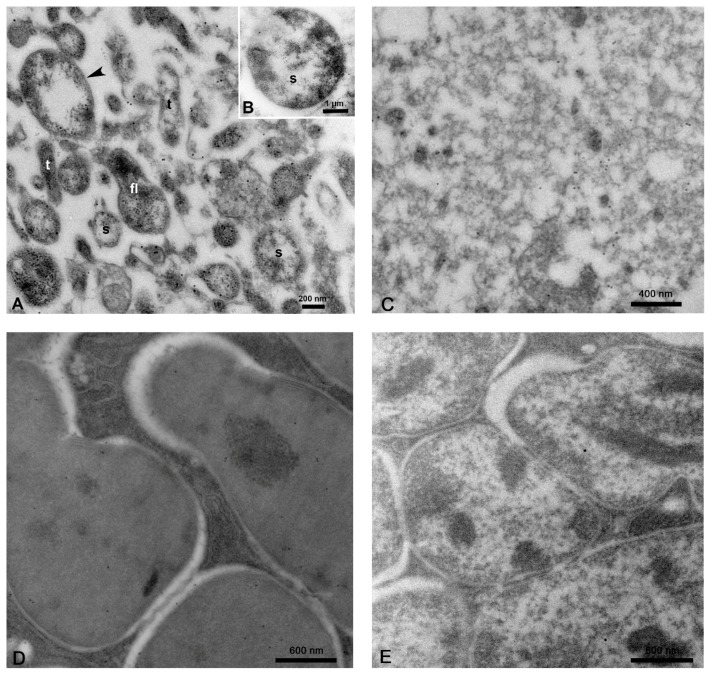
Bacterial cells in tissues and organs of *Candidatus* Liberibacter asiaticus (CLas)-infected *Diaphorina citri* adults, immuno-gold labeled with CLas-OmpA antibodies. (**A**,**B**) Labeled bacterial cells in the midgut are bound by a double membrane (arrowhead) and were either tubular/filamentous (t), spherical/quasi-spherical (s), or flask-shaped (fl). The boxed area in panel B shows an example of a very large CLas cell. (**C**) Background gold levels of the midgut tissue in the absence of CLas. (**D**,**E**) Immuno-gold labeling of endosymbionts in the bacteriome. Both *Carsonella* (**D**) and *Profftella* (**E**) only display background gold levels. (A–E, non-osmicated tissues).

**Figure 2:**
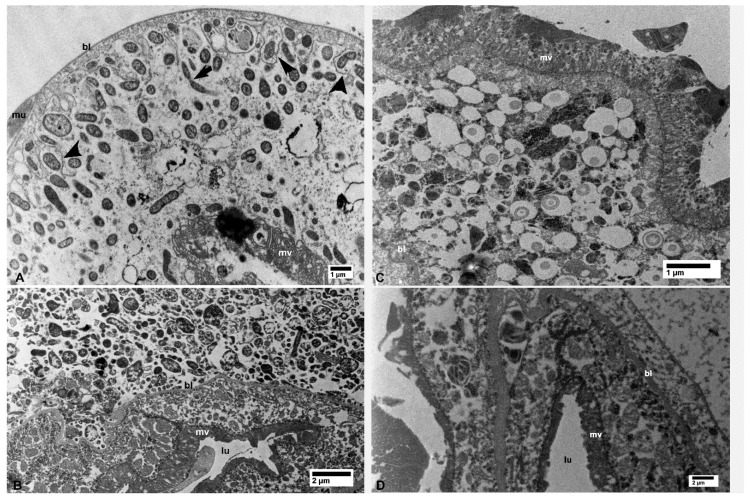
Bacterial cells in the midgut (**A**) and filter chamber (**B**) of CLas-infected *D. citri*. A. In the midgut, bacterial cells are within the vacuolated areas either free in the cytoplasm or located between the infoldings of the basal plasma membranes (arrowheads); the arrow indicates an apparently dividing bacterial cell. (B) Large aggregates of bacterial cells outside the midgut basal lamina (bl), within the filter chamber (top half of the figure, above the basal lamina). No similar bacterial cells were found in the uninfected midgut (**C**) and filter chamber (**D**). Abbreviations: bl—basal lamina; lu—midgut lumen; mu—muscles; mv—microvilli. (**A**: osmicated tissue; **B**–**D**: non-osmicated).

**Figure 3:**
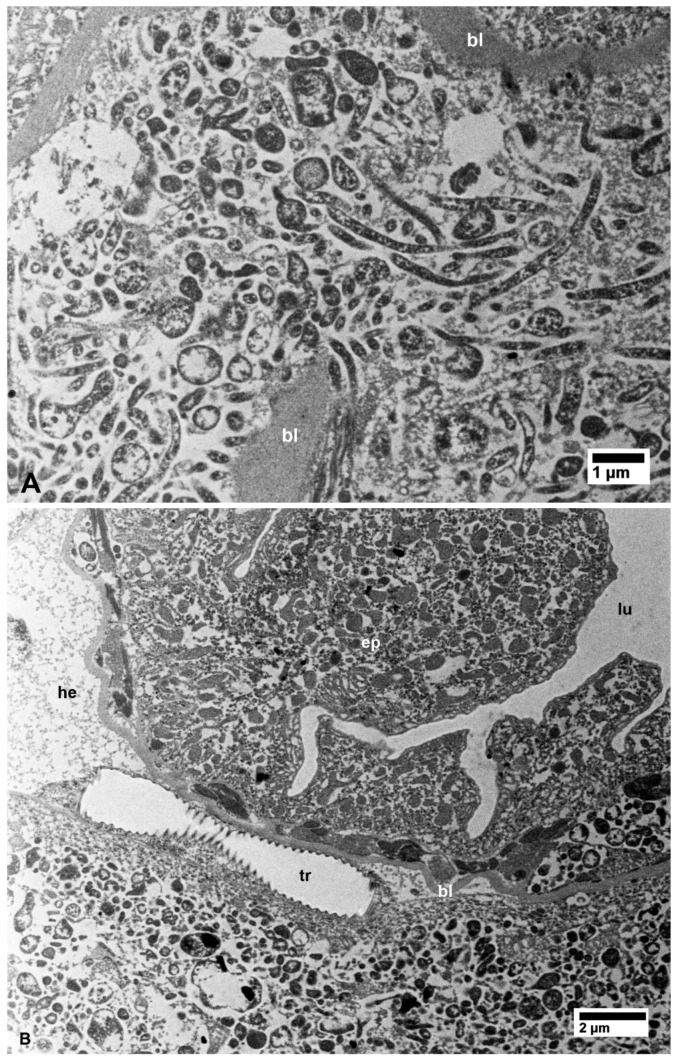
(**A**) Details of bacterial aggregates outside the midgut within the filter chamber of CLas-infected *D. citri*. (**B**) Aggregates of bacteria inside epithelial cells (ep) of the hindgut and outside the hindgut basal lamina (bl) in CLas-infected *D. citri*. Abbreviations—bl, basal lamina; ep—epithelial cells of the hindgut; he—hemocele; lu—hindgut lumen; tr—trachea. (**A** and **B**: non-osmicated tissues).

**Figure 4:**
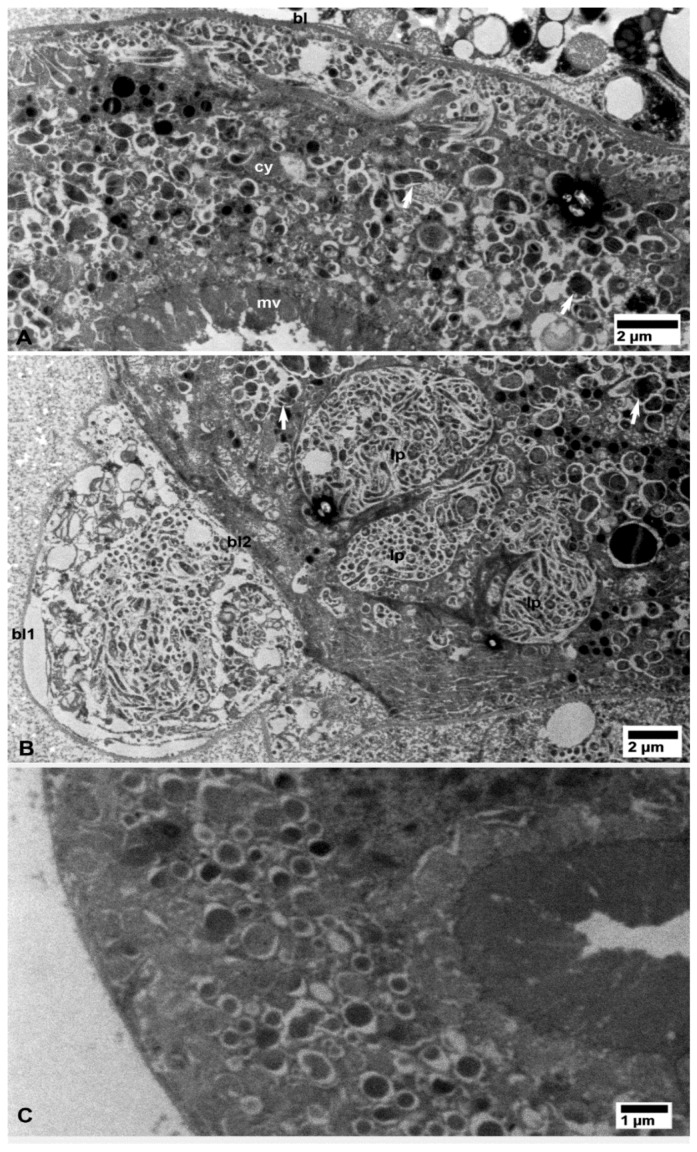
Bacterial cells in the Malpighian tubules of CLas-infected (**A**,**B**) *D. citri*. (**A**) Aggregates of bacterial cells under the basal lamina (bl) and in small pockets in the cytoplasm (cy). (**B**) Bacterial cell aggregations between the two layers of the basal lamina (bl1, bl2) as well as within large pockets (lp) in the cytoplasm. Arrows indicate putative bacterial multiplication sites(also shown below in other organs at higher magnifications). No bacterial cells aggregates were found in similar tissues from healthy control psyllids (**C**). Other abbreviations: mv—microvilli; bl—basal lamina; and cy—cytoplasm. (**A**–**C**: non-osmicated tissues)**.**

**Figure 5:**
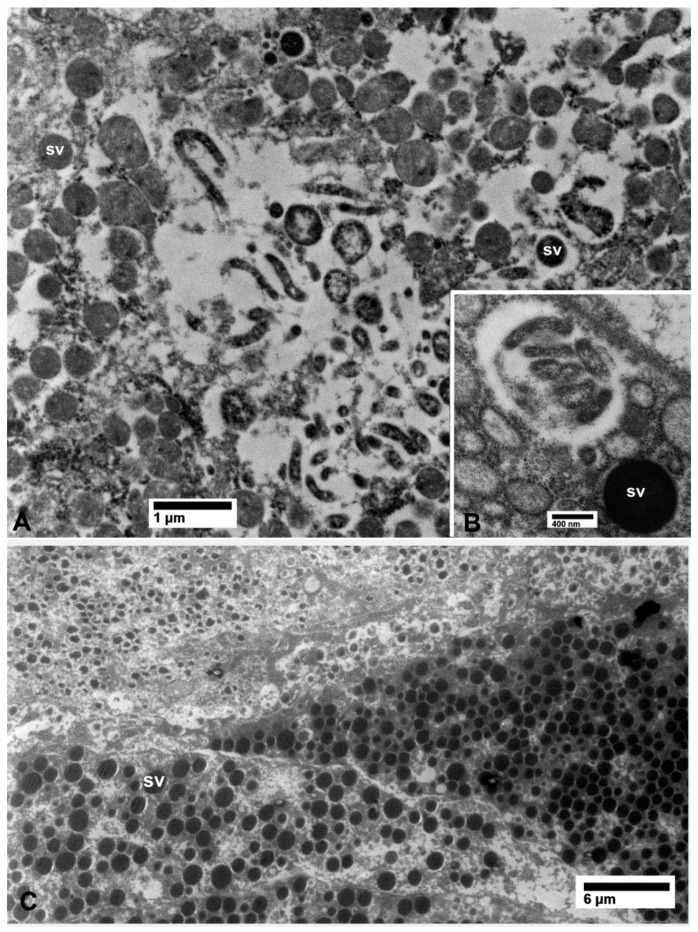
Bacterial cells in the secretory (acinar) cells of the salivary glands of CLas-infected (**A**,**B**) *D. citri*. No bacterial cells were found in similar tissues from healthy control psyllids (**C**) sv, which are the secretory vesicles in the salivary gland cells. (**A**,**C**: non-osmicated tissue, **B**: osmicated).

**Figure 6:**
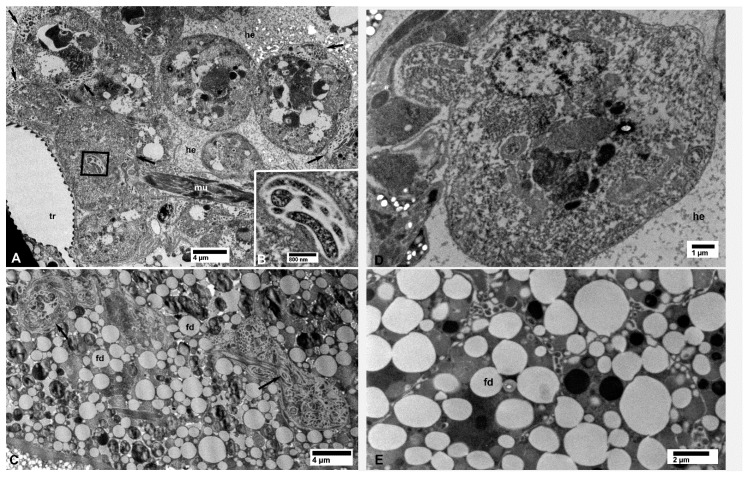
Bacterial cells inside hemocytes within the hemocele (**A**,**B**), and in fat cells (**C**) of CLas-infected (**A**–**C**) *D. citri*. (B) shows a higher magnification of the boxed area in (A); arrows in (C) indicate small pockets of bacteria in fat cell. No bacterial cells were found in the hemocytes (**D**) and the fat cells (**E**) from healthy control psyllids. Abbreviations: fd—fat droplets; he—hemocele; mu—muscle fibers; and tr—trachea. (**A**–**E**, non-osmicated tissues).

**Figure 7:**
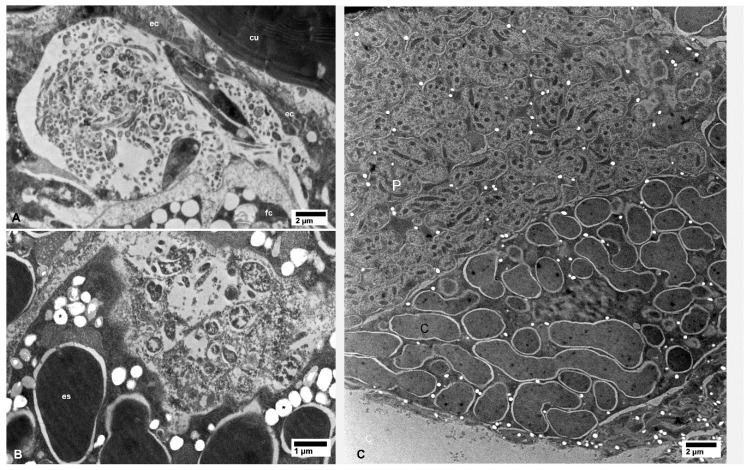
Bacterial cells in epidermal cells of the leg (**A**) and in the bacteriome (**B**) of CLas-infected (**A**,**B**) *D. citri*; asterisks in (B) indicate artifact-holes in the section due to unknown causes. Only *Carsonella* (c) and *Profftella* (p) cells were found in the bacteriome (**C**) from healthy control psyllids. Abbreviations: cu—cuticle; ec—epidermal cell; es—endosymbiont; fc—fat cell. (**A**–**C**: non-osmicated tissues).

**Figure 8:**
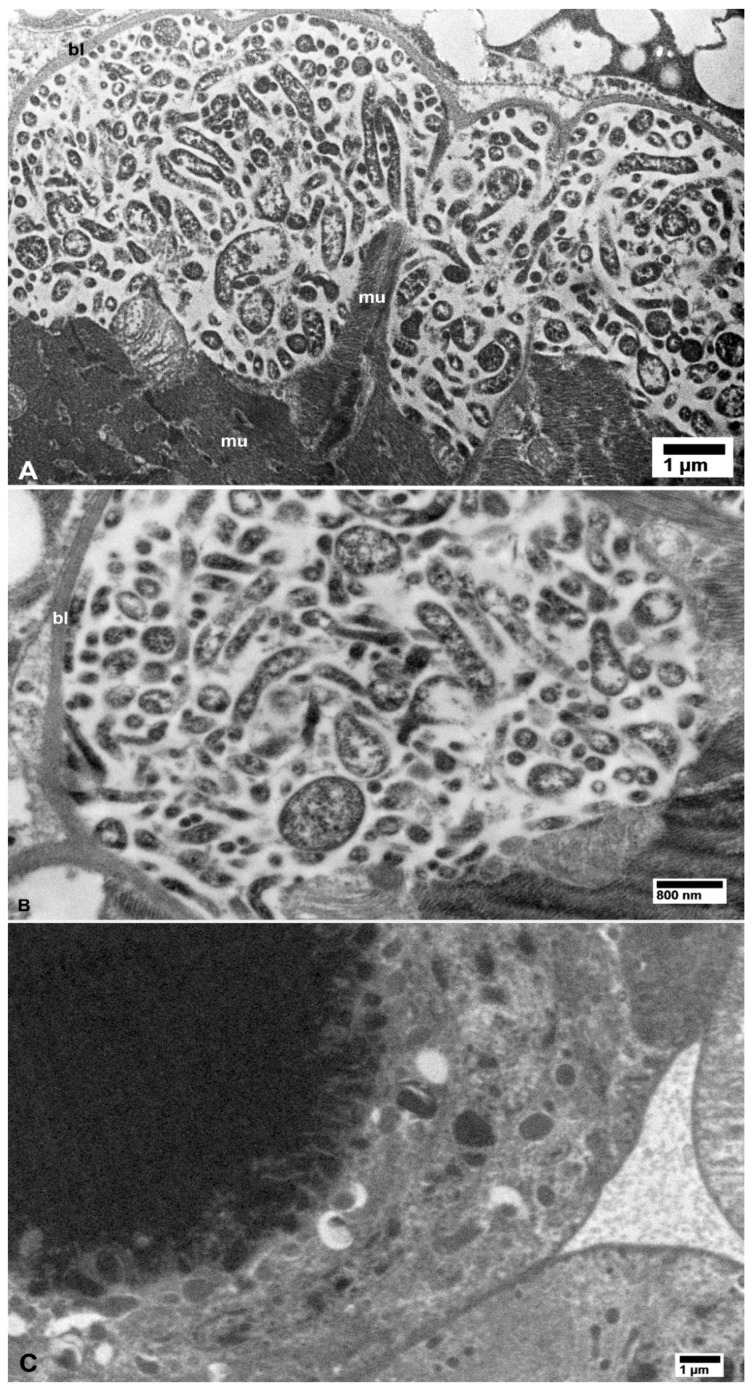
Bacterial cells in muscle tissues of the spermatheca (**A**) and in the oviduct wall (**B**) in CLas-infected *D. citri* females. No bacterial cells were found in the spermatheca from healthy control psyllids (**C**) Abbreviations: bl—basal lamina and mu—muscle fibers. (**A**–**C**: non-osmicated tissues).

Ultrathin sections of the midgut and other organs of CLas-exposed ACP were immuno-gold labeled with antibodies prepared against the OmpA of CLas bacterium. Gold-labeled bacterial cells were pleomorphic (Figure 1A,B), taking different shapes in these sections, including the following—(i) tubular, rod-shaped, or filamentous cells with an average diameter of 183 ± 20 nm reaching an average length of 623 ± 52 nm (N = 41); (ii) spherical or quasi-spherical cells averaging 172.6 ± 5.7 nm in diameter (N = 129); (iii) larger spherical cells averaging 542 ± 22.4 nm in diameter (N = 60), but occasionally reaching 5 µ (Figure 1B); and (iv) flask-shaped cells (Figure 1A) with a spherical base (277 ± 10 nm in diameter) with a tubular tip (133.3 ± 7 nm in diameter) and varying length (N = 13). Each bacterial cell had a double-membrane (arrowheads in Figure 1A) surrounding a nuclear area in the middle. The tubular and small spherical cells were the most abundantly found in most of the tissues examined (Figure 1, Figure 2, Figure 3, Figure 4, Figure 5, Figure 6, Figure 7 and Figure 8). In the absence of CLas, the tissues exhibited a very low and nonspecific background labeling (Figure 1C). To verify that OmpA was specific to CLas in the insect, we also performed immune-gold labeling for the endosymbionts, employing the same protocol. With both *Carsonella* (Figure 1D) and *Profftella* (Figure 1E), only the background gold levels were detected. Overall, no specific labeling was found in the cytoplasm where no Clas bacteria were present (Figure 1C) or on the symbiotic bacteria in the bacteriome (Figure 1D,E).

### 3.2. Ultrastructural Localization of CLas in ACP Organs and Tissues

In epithelial cells of the midgut, outside the filter chamber in CLas-exposed ACP, CLas bacterial cells were abundantly found scattered in vacuolated areas in the cytoplasm, unbound by membranes, either individually or in small aggregates (Figure 2A). CLas cells were also found abundantly under the basal lamina in the basal part of epithelial cells, in vacuolated areas that were partially surrounded by the basal plasma membranes (arrowheads in Figure 2A). Some of the tubular-shaped cells appeared to be dividing (arrow in Figure 2A).

In the filter chamber of CLas-exposed ACP, small aggregates of CLas cells were similarly found in the cytoplasm of the midgut epithelial cells, under the basal lamina (bl in Figure 2B). However, much larger aggregates of CLas cells were found outside the midgut basal lamina, but still inside the filter chamber (Figure 2B and Figure 3A). Similar aggregates were found outside the hindgut (bl in Figure 3B) between the sublayers of the basal lamina, in addition to smaller aggregates found in the cytoplasm of the hindgut epithelial cells (ep in Figure 3B). In the Malpighian tubules outside the filter chamber, small aggregates of CLas were found in the cytoplasm, under the basal lamina (bl in Figure 4A), and much larger aggregates were found between the sublayers of the basal lamina (bl1 and bl2 in Figure 4B), or in large pockets in the cytoplasm (lp in Figure 4B).

**Figure 9:**
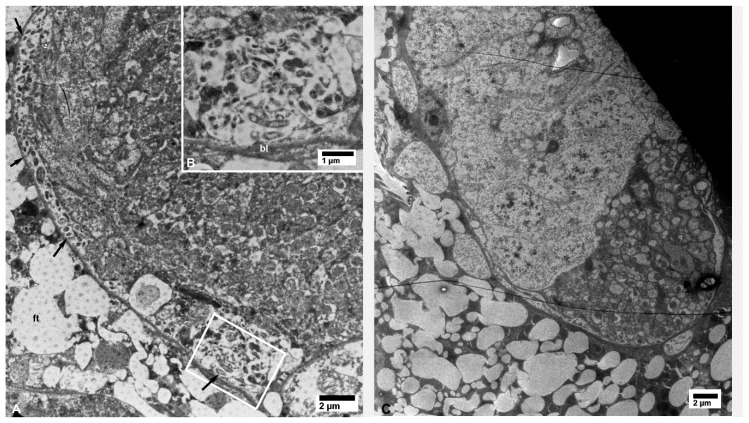
Small aggregates of bacterial cells (arrows) under the basal lamina (bl) of neural tissues in CLas-exposed *D. citri* (**A**,**B**); the boxed area in panel (A) is shown at a higher magnification in panel (B). No bacterial cells were found in similar tissues from healthy control psyllids (**C**); ft—fat tissue.

Large or small aggregates of CLas cells were also found in the vacuolated areas in the cytoplasm of secretory cells of the salivary glands in the CLas-exposed psyllids (Figure 5A,B), in hemocytes within the haemocoele (arrows in Figure 6A), in fat cells (arrows in Figure 6C), and in epidermal cells under the cuticle of many structures including a leg (Figure 7A). Aggregates of the CLas cells were found within cells of the bacteriome, adjacent to other (much larger) bacterial symbionts (‘es’ in Figure 7B). In CLas-exposed female psyllids, large aggregates of CLas cells were found under the basal lamina of the muscles surrounding the spermatheca (Figure 8A) and in the oviduct epithelial wall (Figure 8B). In neural tissues of CLas-exposed ACP, small aggregates of CLas cells were found under the basal lamina of large nerves (Figure 9A,B) close to the compound ganglionic mass, which is located in the thorax under the salivary glands.

No CLas cells were found in cell nuclei of CLas-exposed ACP organs/tissues, or in any of the healthy controls (Figure 1, Figure 2, Figure 3, Figure 4, Figure 5, Figure 6, Figure 7, Figure 8 and Figure 9). Additionally, apart from the above-mentioned accumulations of CLas bacteria in the cytoplasm, no remarkable cytopathological changes were observed in various tissues of CLas-infected ACP adults, compared to those of unexposed healthy control ones (Figure 2, Figure 3, Figure 4, Figure 5, Figure 6, Figure 7, Figure 8 and Figure 9).

### 3.3. Putative Multiplication Sites of CLas

**Figure 10:**
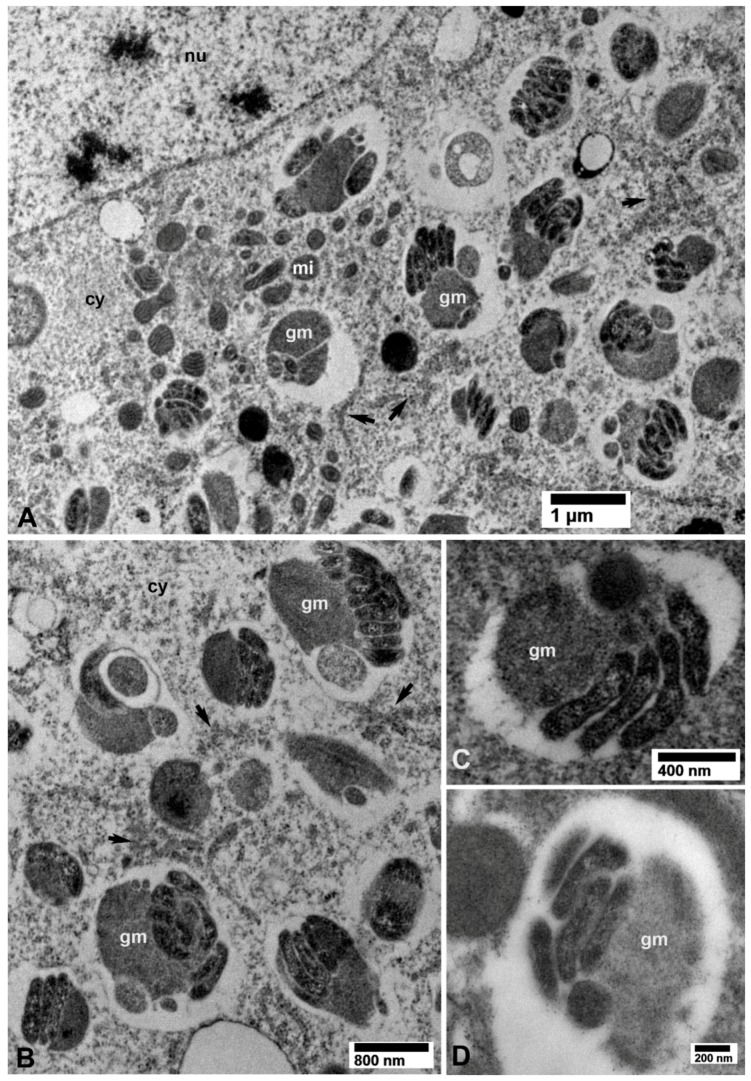
Putative CLas multiplication sites containing electron-dense granular matrix (gm) and closely packed bacterial cells, within vacuolated areas in the cytoplasm, in the vicinity of rough endoplasmic reticulum (arrows), in epithelial cells of the midgut (**A**,**B**), fat tissue (**C**), and the salivary gland (**D**) in CLas-exposed *D. citri*. Abbreviations: cy—cytoplasm; gm—granular matrix; mi—mitochondria; and nu—nucleus. (**A**–**D**: osmicated tissues).

**Figure 11:**
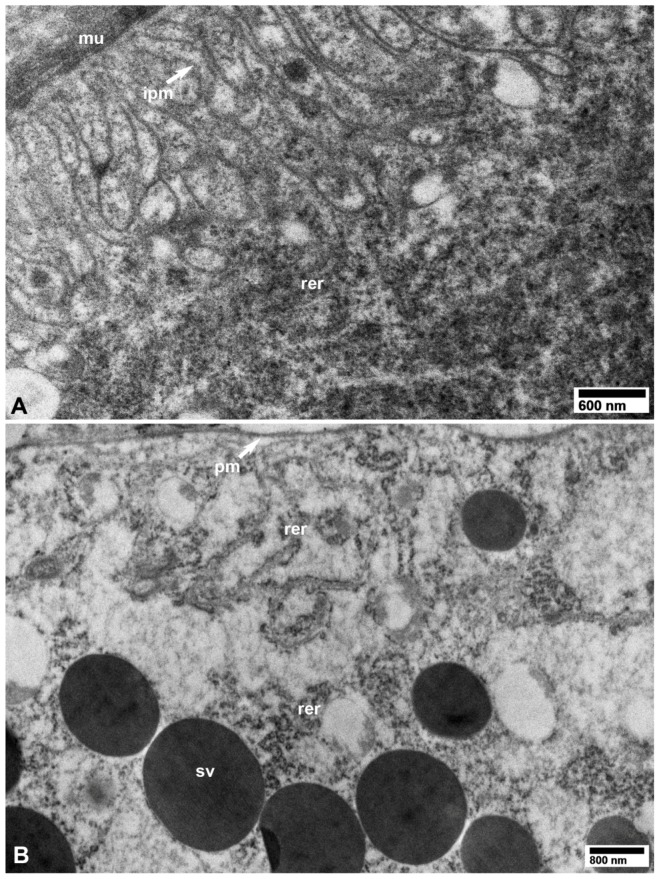
TEM images from healthy control *D. citri* adults (unexposed to CLas). (**A**) Basal part of a midgut epithelial cell, showing surrounding muscle fibers (mu), infoldings of the basal plasma membrane (ipm), and rough endoplasmic reticulum (rer). (**B**) Part of a secretory cell in the salivary gland showing plasma membrane (pm), secretory vesicles (sv), and rough endoplasmic reticulum (rer). (**A**,**B**: osmicated tissues).

In several tissues from the CLas-exposed ACP, non-membrane bound intracytoplasmic vacuolated areas, distinct from the above-described bacteria-containing ones, enclosed closely packed bacterial cells that are usually associated with electron-dense granular matrix (gm in Figure 10A,B) as well as loosely arranged rough endoplasmic reticulum (RER, arrows in Figure 10A,B). These structures were found in CLas-exposed ACP colonies from both Lake Alfred (Figure 4) and Fort Pierce (Figure 10). We hypothesized that these intracytoplasmic inclusions were multiplication sites of the CLas bacterium (see further details in the discussion). These putative multiplication sites were found in the epithelial cells of the midgut (Figure 10A,B), Malpighian tubules (Figure 4), salivary gland secretory cells (Figure 5B and Figure 10D), and in fat cells (Figure 10C). These putative multiplication sites, as well as the other intracytoplasmic CLas-containing inclusions described above, were found only in CLas-exposed ACP adults (Figure 2, Figure 3, Figure 4, Figure 5, Figure 6, Figure 7, Figure 8, Figure 9 and Figure 10), but not in the non-exposed healthy control ones (Figure 2, Figure 3, Figure 4, Figure 5, Figure 6, Figure 7, Figure 8 and Figure 9 and Figure 11).

## 4. Discussions

Localization of bacteria-like structures, associated with huanglongbing/citrus greening, in the salivary glands and gut of infected ACP and the African citrus psyllid, Trioza erytreae (Hemiptera: Triozidae) has been reported earlier in China and South Africa, respectively, using thin-sectioning electron microscopy [14,15,21]. However, no immunolabeling or molecular labeling methods were available to confirm the identity of such bacteria in these studies. Later, using fluorescent in situ hybridization (FISH) or qPCR indicated the presence of CLas in most organs and tissues of CLas-infected *D. citri* adults from Florida [6,7,10,22]. More recently, Ghanim et al. [16] used FISH and immunogold labeling to describe CLas accumulation in the cells of ACP midgut. However, our current study, supported by immunogold labeling, provides the most comprehensive ultrastructural study of the cellular and subcellular localization of CLas in ACP vector cells, organs, and tissues, till date.

In infected ACP tissues, CLas cells were pleomorphic, taking tubular, quasispherical, or flask-shaped forms, some of which seemed to be dividing further. At least some of the small spherical cells could be cross sections of the tubular-shaped ones, whereas the flask-shaped cells might represent a transition from the spherical to the tubular-shaped cells during bacterial growth or multiplication. We believe that these various forms are part of CLas pleomorphic life cycle during growth or multiplication of this bacterium in the vector, as is the case with some of the hemipteran-borne phytopathogens in the genus *Spiroplasma* (Class Mollicutes). Spiroplasmas are also pleomorphic and the same species might have helical, tubular, round, flask-like, or other irregular shapes [23]. In general, extracellular *Spiroplasma* cells are more frequently helical, whereas spiroplasmas that are located intracellularly are more often round or flask-shaped [24,25]. However, with CLas, both the tubular and quasispherical forms were abundant intracellularly in the cytoplasm of infected ACP cells. No CLas cells were found extracellularly in infected ACP tissues, except for the large accumulations of Class cells between layers or sublayers of the basal lamina outside the midgut within the filter chamber and outside the hindgut and Malpighian tubules. These CLas accumulations are somewhat similar to, but less extensive than, the bacterial biofilms that were also reported outside the midgut, within the filter chamber of the potato psyllid vector of the zebra chip pathogen CLso [11]. This is in line with previous reports indicating that transmission efficiency and CLso titer in infected potato psyllids [26,27] is usually much higher than those of CLas in infected ACP [7,28,29].

In our study, the near systemic invasion of CLas in most of the ACP organs and tissues examined, including the epidermis of a leg (Figure 7A), supported previous FISH and qPCR studies indicating a wide distribution of CLas in infected ACP [5,6,10,22,29], as well as ultrastructural, qPCR and FISH studies that have indicated an almost systemic invasion of the zebra chip pathogen CLso in the potato psyllid vector [11,27,30]. The near systemic distribution of both Liberibacter pathogens in their psyllid vectors was similar to that of other circulative-propagative hemipteran born bacterial phytopathogens, viz. *Spiroplasma* and Phytoplasma, in their leafhopper vectors [23,31]. We found small aggregates of CLas under the basal lamina of neural tissues near the compound ganglionic mass located under the salivary glands [13]; this has not been reported earlier. With CLas, CLso, *Spiroplasma*, and Phytoplasma, the lack of obvious infections in cell nuclei or extensive infection of the nervous system of the psyllid or leafhopper vectors might indicate a long co-evolutionary adaptation where the pathogen can be transmitted by these obligate vectors, without substantially harming them, which confers a survival advantage to these phytopathogens. In this regard, CLas-infected ACP exhibited a higher finite rate of population increase and net reproductive rate, although ACP nymphs and adults had a reduced longevity, as compared to uninfected ACP [32,33,34].

The ‘putative multiplication sites’ for CLas bacteria reported here in several infected ACP tissues, characterized by the inclusion of a granular matrix with closely packed bacterial cells, has not been reported earlier with CLas or CLso in their psyllid vectors, but it is reminiscent of the ‘viroplasm’ inclusions or ‘virus factories’, reported with some animal and plant-pathogenic reoviruses, e.g., rice dwarf phytoreovirus transmitted by leafhoppers [35]. The association of these putative CLas multiplication sites with RER, lends support to a previous study by Ghanim et al. [16] who reported that CLas induced the formation of endoplasmic reticulum (ER)-associated bodies in the gut. They suggested that CLas might recruit those ER-structures into Liberibacter-containing vacuoles (LCVs), in which the bacterial cells seem to propagate. Interestingly, ER-associated LCV formations were not detected in the potato psyllid infected with CLso [16].

These putative CLas multiplication sites, in addition to the apparently dividing CLas cells and the near systemic distribution of CLas in infected ACP cells and tissues, support the circulative–propagative mode of transmission for CLas in its ACP vector, as suggested earlier [4,7]. However, it is not clear how CLas or CLso moves from the midgut into the hemocele or other organs, or how they move from these to the salivary gland through the presumed entry or exit barriers, e.g., microvillar and plasma membranes or basal lamina. Specific mechanisms mediating Liberibacter recognition, attachment, and multiplication in vector organs are not yet clear [36]. To elucidate these important events, further investigations using TEM, immunolabeling or molecular labeling, and involving a time-course study of vector psylllids post-CLas or -CLso acquisition while they feed on infected plants are required. The present work, as well as similar TEM investigations reported earlier [11,13], can pave the way for such future studies to understand the molecular and cellular interactions leading to acquisition, transmission, and vector competence of these economically important pathogens by their respective vectors. These studies might also enable us in the future to devise novel and more ecofriendly control methods for these devastating plant pathosystems.
